# Symptom-driven inhaled corticosteroid/long-acting beta-agonist therapy for adult patients with asthma who are non-adherent to daily maintenance inhalers: a study protocol for a pragmatic randomized controlled trial

**DOI:** 10.1186/s13063-022-06916-3

**Published:** 2022-12-05

**Authors:** James G. Krings, Kaitlyn M. Wojcik, Vanessa Chen, Tejas C. Sekhar, Kelly Harris, Abigail Zulich, Kaharu Sumino, Ross Brownson, Eric Lenze, Mario Castro

**Affiliations:** 1 Division of Pulmonary and Critical Care Medicine, Washington University in Saint Louis School of Medicine, Saint Louis, MO 63110 USA; 2 Brown School of Social Work and Public Health, Washington University in Saint Louis, Saint Louis, MO 63110 USA; 3 Program in Occupational Therapy, Washington University in Saint Louis School of Medicine, Saint Louis, MO 63110 USA; 4 Division of Psychiatry, Washington University in Saint Louis School of Medicine, Saint Louis, MO 63110 USA; 5 Division of Pulmonary Critical Care and Sleep Medicine, University of Kansas, Kansas City, KS 66103 USA

**Keywords:** Asthma, Inhaler, Medication adherence, Pragmatic clinical trial, Qualitative

## Abstract

**Background:**

While inhaled corticosteroids (ICS) are considered the essential foundation of most asthma therapy, ICS inhaler nonadherence is a notoriously common problem and a significant cause of asthma-related morbidity. Partially acknowledging the problem of nonadherence, international organizations recently made paradigm-shifting recommendations that all patients with mild-to-moderate persistent asthma be considered for symptom-driven ICS-containing inhalers rather than relying on adherence to traditional maintenance ICS inhalers and symptom-driven short-acting beta-agonists (SABA). With this new approach, asthma patients are at least exposed to the important anti-inflammatory effects of ICS-containing inhalers when their symptom reliever inhaler is deployed due to acute symptoms.

**Methods:**

This study will (Part 1) complete a pragmatic randomized controlled trial to evaluate if an inhaler strategy that utilizes symptom-driven ICS inhalers is particularly beneficial in maintenance ICS inhaler non-adherent asthma patients, and (Part 2) use a dissemination and implementation (D&I) science conceptual framework to better understand patients’ and providers’ views of inhaler nonadherence. This study, which will have an option of taking place entirely remotely, will use a Food and Drug Administration (FDA)-approved electronic sensor (Hailie® sensor) to monitor inhaler adherence and includes semi-structured interviews guided by the Consolidated Framework for Implementation Research (CFIR).

**Discussion:**

This study is assessing the problem of nonadherence using a D&I implementation science research lens while testing a new inhaler approach to potentially ameliorate the detrimental consequences of maintenance inhaler nonadherence. We hypothesize that the use of a symptom-driven ICS/LABA management strategy, as compared to traditional maintenance ICS treatment and symptom-driven SABA, will lead to improved adherence to an asthma treatment strategy, decreased asthma-related morbidity, less cumulative ICS exposure, and greater patient satisfaction with an inhaler approach.

**Trial registration:**

ClinicalTrials.gov NCT05111262. Registered on November 8, 2021.

**Supplementary Information:**

The online version contains supplementary material available at 10.1186/s13063-022-06916-3.

## Introduction

### Background

#### Asthma Morbidity and Consequences of Inhaler Nonadherence

Asthma is a common disease characterized by chronic airway inflammation, variable airflow obstruction, and recurrent respiratory symptoms that affect >300 million people worldwide [1–3]. In the United States (US), >50% of patients with asthma are uncontrolled, annually resulting in approximately 2 million emergency department visits, and >3500 deaths in the US [4–9]. The reasons many patients with asthma remain uncontrolled are multifactorial and complex; however, low adherence to prescribed inhaler regimens is notoriously common and a major contributor to asthma-related morbidity [10–14]. Irrespective of how inhaler adherence is measured (electronic inhaler monitors, prescription claims data, or self-report), studies have consistently shown that 40–80% of patients with asthma are nonadherent to their prescribed maintenance inhaler regimen [14–16].

The reasons for maintenance inhaler nonadherence are complicated and variable, but often attributed to the beliefs patients have regarding their asthma condition itself and its treatments. Many patients believe that they “only have asthma” when they are experiencing symptoms, and only decide to to adhere to their medication regimen if they deem it necessary (i.e., only when they are symptomatic) [17, 18]. Furthermore, it is also important to note that discordance in perceptions and expectations of asthma control between healthcare providers and patients can also be a factor affecting treatment choices and adherence [19, 20]. For example, a provider may only deem a patient under control if exacerbations are altogether prevented and progressive lung function decline normalized. Conversely, a patient may deem his or her asthma to be under control if they do not have daily symptoms (even though they are having intermittent exacerbations that put them at risk of a serious detrimental event).

Since asthma is characterized by chronic airway inflammation and subsequent long-term airway remodeling, ICS therapy is considered the essential cornerstone of asthma therapy as it ameliorates airway inflammation. Poor adherence to maintenance ICS therapy has been previously associated with an increased frequency of asthma exacerbations, accelerated longitudinal lung function decline, a greater number of missed school and workdays, asthma-related hospitalizations, and asthma-related death [10, 21–28]. Prior studies have estimated that 25% of asthma exacerbations and 60% of asthma-related hospitalizations could be avoided with even reasonable ICS adherence [10, 14–16, 29].

### Rationale for this study

#### Update in 2019 asthma recommendations

US and international asthma guidelines have traditionally recommended daily maintenance ICS therapy for all patients with asthma except for those with only intermittent disease [30, 31]. However, the Global Initiative of Asthma (GINA) and the National Institutes of Health (NIH)’s National Asthma and Education Program (EPR-4) recently issued paradigm-shifting strategy reports, which finally acknowledged that patients in real-world settings are often nonadherent to their daily ICS inhalers [3, 32]. In the most recent strategy reports, GINA and EPR-4 recommended that all adults with mild persistent asthma be considered for symptom-driven ICS/long-acting beta-agonist (LABA) inhalers rather than relying on daily maintenance ICS inhalers (which many patients frequently do not take anyways) and symptom-driven SABAs [3, 32]. Evidence supporting this approach comes from recent clinical trials that demonstrated the clinical equipoise between a symptom-driven ICS/LABA treatment strategy and traditional therapy [33–36]. However, prior studies have opined that providers are unaware of or skeptical of these latest recommendations [37, 38].

#### Rationale for use of ICS/LABA in maintenance inhaler non-adherent patients

We believe that if adults with mild persistent asthma who are nonadherent to their maintenance ICS inhaler were identified in clinical practice, switching these patients to a symptom-driven ICS/LABA inhaler approach may be more beneficial than leaving them on their traditional therapy plan. The rationale for this proposition can be partially observed from the results of the recent SYGMA-1 trial [33]. In this study, patients ≥12 years old with mild asthma were randomized to one of three regimens: twice-daily maintenance ICS plus SABA as-needed (budesonide group), twice-daily placebo plus SABA as-needed (terbutaline group), or twice-daily placebo plus ICS/LABA as-needed (budesonide/formoterol group). Patients in the budesonide/formoterol group as compared to the terbutaline group had better outcomes and a markedly prolonged time to first asthma exacerbation (hazard ratio, 0.44; 95% CI 0.33 to 0.58). One could wonder if an adult with asthma who is nonadherent to their maintenance inhaler (and thus only using a SABA as needed like the terbutaline group above), could experience a marked reduction in their exacerbation risk if their rescue inhaler was simply switched to an ICS/LABA (similar to what the budesonide/formoterol group was receiving). Such therapeutic changes have never been intentionally made as a way to tackle maintenance inhaler nonadherence specifically.

In prior focus groups, described reasons for nonadherence to maintenance inhalers were diverse and included: forgetfulness, a belief that taking a steroid-containing inhaler when feeling well is potentially harmful, and that maintenance inhalers are simply unnecessary when feeling well [26]. If adults who were nonadherent to maintenance ICS inhalers were identified, providers could potentially change their rescue SABA to a rescue ICS/LABA and instruct patients to use their inhalers as they already are (only actuating an inhaler when symptomatic, which does not require remembering to deploy an inhaler when feeling well) and potentially improve asthma outcomes [39–41].

### Study objectives

*Part 1:* To evaluate if a strategy that utilizes symptom-driven ICS/LABA inhalers (new approach), as compared to maintenance ICS and symptom-driven SABA inhalers (traditional approach), improves adherence to a treatment strategy and improves asthma-related morbidity in adult patients who were previously non-adherent to maintenance ICS inhalers.

We will conduct a pragmatic, open-label, randomized control trial that can be implemented remotely with 50 mild-to-moderate persistent asthma patients identified as nonadherent to their maintenance ICS inhalers. After a pre-randomization run-in period, participants will be randomized to either a symptom-driven ICS/LABA treatment strategy or continue their current daily ICS and symptom-driven SABA regimen. The primary outcome will be adherence to a management strategy, which will be assessed via an FDA-approved electronic inhaler sensor (Hailie® device) and defined as the proportion of ICS-containing inhaler actuations per strategy recommendation. Secondary outcomes that will be assessed include time to first study-defined asthma exacerbation, number of asthma exacerbations, change in asthma morbidity based on validated questionnaires, cumulative ICS exposure, self-efficacy, and patient satisfaction with a treatment strategy.

*Part 2*: To assess the facilitators and barriers within adult asthma patients and their providers to (1) improving inhaler adherence and (2) utilizing the latest recommendation of a symptom-driven ICS/LABA treatment strategy.

As a key pre-implementation step, we will conduct semi-structured interviews with a diverse group of maintenance inhaler non-adherent patients and their providers to determine the facilitators and barriers to inhaler adherence and the use of the latest recommendations for a symptom-driven ICS/LABA treatment strategy. These semi-structured interviews will be guided by CFIR. Interviews will be recorded, categorized, and conducted with the goal of reaching theme saturation.

## Methods

### Overview and design summary

This is a two-part study that involves both qualitative and quantitative assessments. Part 1 of this study is a pragmatic randomized controlled trial. Participants that completed Part 1 of the study and providers will be invited to complete a post-study qualitative assessment in Part 2 of the study. This methods section followed SPIRIT reporting guidelines [40].

#### Part 1: pragmatic randomized controlled trial methods

**Fig. 1:**
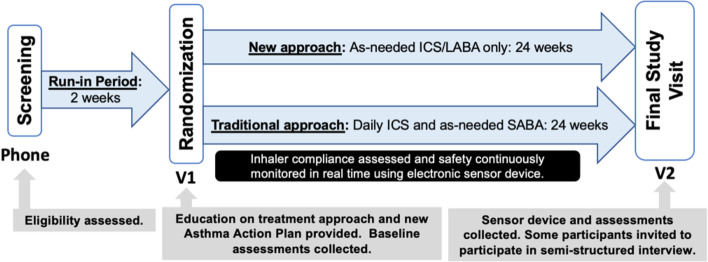
Study design. Participants will complete a 2-week period wherein compliance with smartphone questionnaires will be confirmed. This will be followed by randomization and 24 weeks of treatment before a final study visit

In this study, we are conducting a pragmatic, open-label, 2-arm, randomized control trial of 50 maintenance inhaler-non-adherent asthma patients (with 25 participants receiving a new symptom-driven ICS/LABA treatment strategy and 25 continuing maintenance ICS and SABA therapy; Fig. 1). Identified participants in Barnes-Jewish Hospital (BJH) or Washington University in St. Louis (WUSTL) affiliated clinics will be contacted and screened for eligibility over the phone using a recruitment script. Additionally, advertisements for this study will be placed in approved areas on the medical campus. If participants are eligible and willing, twice daily text messages will be sent with their permission to their phone to confirm their ability to complete twice-daily assessments of their asthma control via REDCap texting to their phone for 2 weeks [41, 42]. Permission for this text assessment will be obtained on the phone only. No other interventions whatsoever (including changes in treatment) will take place until written consent is obtained at the first in-person or Zoom-based study visit. Patients who do not complete at least 70% (or 20/28) of these one-item text assessments will not be invited for the first comprehensive study visit and their participation in the study will be ended. Interested and eligible participants will then complete visit 1 (V1) either via Zoom or in our clinical research space. If potential participants elect to perform their visit on Zoom, we will recommend they seek a quiet private environment, and the process will be similarly treated between the potential participant and research staff as would be the case in-person. The decision was made to add a Zoom-only option for study visits to decrease study burden on asthma patients — particularly considering the ongoing COVID-19 pandemic. The informed consent process will utilize the identical approved informed consent document in REDCap (or via paper and pen if in-person and the participant prefers).

**Fig. 2:**
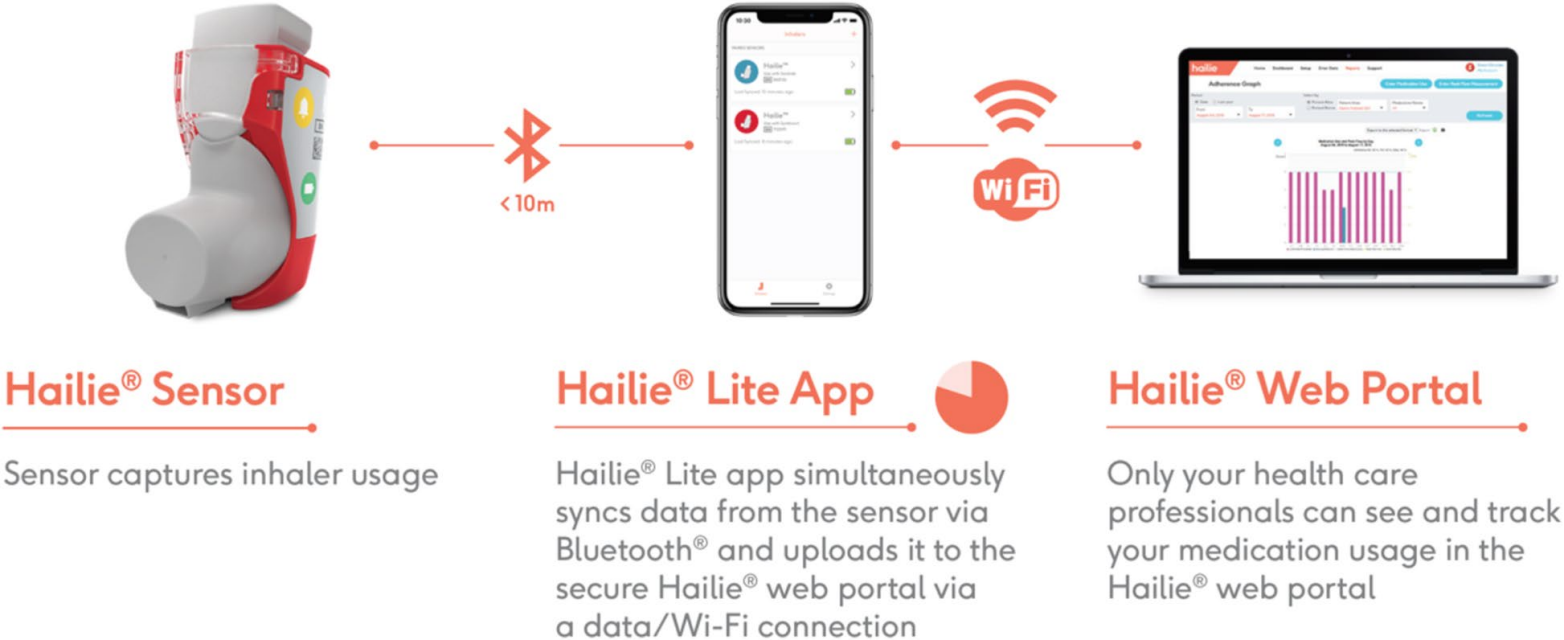
Hailie® Sensor for SYMBICORT pMDI and Hailie® app. *© 2022 Adherium (NZ) Limited*

After informed consent has been completed, participants will receive further characterization with validated questionnaires including the Medication Adherence Reporting Scale for Asthma (MARS-A) questionnaire, medical history questionnaire, Asthma Control Test (ACT), Asthma Quality of Life Questionnaire (AQLQ), Asthma Control Questionnaire (ACQ), IQVIA Treatment Satisfaction Questionnaire for Medication (TSQM), the Knowledge, Attitude, and Asthma Self-Efficacy Asthma Questionnaire (KASE-AQ), the Inhaled Corticosteroid Side-Effect Questionnaire – Brief Version (ICQ-S), the Multidimensional Health Locus of Control Scale (MHLC), and the Feeling of Satisfaction with Inhaler (FSI-10) questionnaire. Participants will then be randomized using a 1:1 ratio with random numbers generated using SAS 9.4 (Cary, NC, USA) to a treatment arm. A new Asthma Action Plan will be created and reviewed with the participants and their medication sent to their pharmacy. All participants will then have a Hailie® sensor attached to their inhalers (if in-person) or sent to their address (if using a Zoom-based visit) (Fig. 2) Once the patient has received their inhaler and sensor, the study team will contact participants to be sure they are comfortable with the set-up process and recommend that they deploy one test inhaler actuation to ensure the sensor is working properly. Of note, the Hailie® device is an FDA-approved electronic inhaler sensor and smartphone application that has been successfully and safely used in other NIH-funded studies to assess inhaler adherence [42–52].

A participant’s primary asthma provider will be informed of their patient’s assigned treatment strategy and documentation of any changes in their treatment will be noted in the electronic medical record if at WUSTL/BJH (documentation of placement of their research involvement in the electronic medical record is noted in the informed consent document in REDCap). In the new approach treatment arm, participants will be instructed to stop using their maintenance ICS inhaler and SABA, and to instead use their new ICS/LABA inhaler, budesonide 160 mcg/formoterol 4.5 mcg, 1 puff as needed for symptoms in a manner similar to how they would have previously been instructed to use their SABA. Budesonide/formoterol was specifically chosen due to its widespread availability, formoterol’s rapid onset of action [53–55], and positive results from prior clinical trials [33–36]. Inhaler adherence will be assessed using the Hailie® sensor, and exacerbation frequency will be collected by surveys texted to a participant’s phone with a REDCap link and via telephone calls conducted every 8 weeks by the study team. After 24 weeks, participants will either return for a final study visit in-person or via Zoom wherein administration of validated baseline questionnaires will be repeated. At that final visit, inhaler sensors will be collected from participants. For participants who elect to do their final visit remotely, an addressed and stamped envelope will be sent to participants in order to return the sensor. Participants will also be invited to participate in a brief exit interview to delineate their thoughts regarding their treatment strategy.

### Outcome measures

#### Primary outcome

**Fig. 3:**
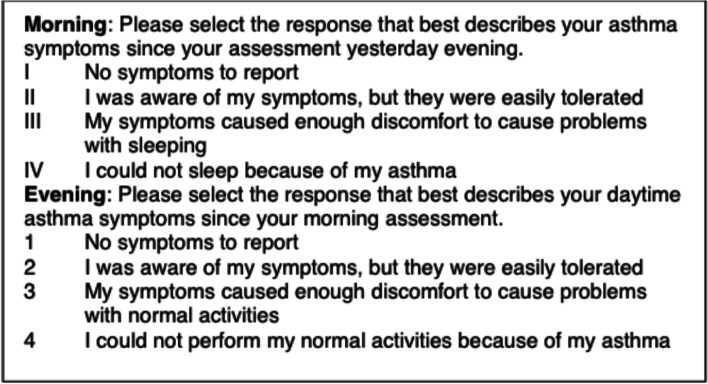
REDCap text-delivered home monitoring of symptoms questions

In Part 1 of the study, the primary outcome will be adherence with an inhaler management strategy delineated using the Hailie® inhaler sensor and smartphone application. In addition, a REDCap survey will be specifically programmed to deliver morning and evening questions to the participants’ smartphone as shown in Fig. 3. This questionnaire to quickly assess symptoms on a mobile device is identical to what is being used in the multicenter NIH-funded Precision Interventions for Severe and/or Exacerbation-Prone Asthma Network (PrecISE) trial (ClinicalTrials.gov identifier: NCT04129931). Adherence to the treatment strategy for the maintenance ICS inhaler group will be calculated as the proportion of maintenance ICS actuations per prescription recommendation. Adherence for the symptom-based ICS treatment strategy will be calculated as the proportion of ICS/LABA actuations per recommendation based on self-report of symptoms (definition: ICS/LABA inhaler actuation during a day or night period divided by a period wherein a patient identified symptoms as ≥2 on REDCap-delivered smartphone questionnaire).

#### Secondary outcomes

Secondary outcomes that will be assessed include the frequency of study-defined asthma exacerbations (adjusted for time on treatment), time to first exacerbation, cumulative dose of ICS, occurrence of study-defined severe and non-severe adverse events and changes in MARS-A questionnaire, medical history questionnaire, ACT, AQLQ, ACQ, IQVIA TSQM, the ICQ-S, the MHLC, KASE-AQ, and FSI-10 questionnaire from baseline to the final study visit. Asthma exacerbations will be defined as an urgent medical visit for asthma symptoms, prescription of oral corticosteroids for asthma symptoms, or high use of a rescue inhaler (>16 actuations of SABA or >8 actuations of ICS/LABA within 24 h on two consecutive days as assessed via the Hailie® device).

### Study treatments

Participants will be randomized to one of two treatment approaches.

#### New approach

Participants will be instructed to use budesonide 160 mcg/formoterol 4.5 mcg, 1 puff as needed for asthma symptoms, which is similar to how most patients would have previously been told to use their rescue SABA (such as albuterol) inhaler. No maintenance inhaler is utilized in this approach.

#### Traditional approach

Participants will be instructed to continue using a maintenance fluticasone inhaler on a standing basis with a SABA as needed. For uniformity and in order to utilize the Hailie® sensor, all participants’ ICS will be changed to fluticasone at a dose that is equivalent to their current ICS prescription using a steroid equivalency chart [56].

### Participant selection and withdrawal

We aim to recruit patients until at least 50 participants have been randomized, successfully picked up their treatment medication, attached the inhaler sensor, and completed >70% of REDCap questionnaires for 2 weeks. We estimate that we will have to randomize approximately 60 (but potentially up to 100) maintenance inhaler non-adherent participants who meet the inclusion/exclusion criteria outlined below in order to achieve this goal. Specifically, we aim to recruit patients who have partially controlled or moderately uncontrolled asthma (wherein as-needed LABA/ICS is advocated for and considered safe). Patients will be recruited from WUSTL’s pulmonary allergy, and primary care clinics, as well as from our posted flyer advertisements. Potential participants will be identified by providers or by review of the electronic medical record and contacted by the study team in-person over the phone using an Institutional Review Board (IRB)-approved phone screening script.

### Inclusion criteria

1. Able to understand and provide informed consent.
2. Age 18–75 at the time of study enrollment.
3. Diagnosed by a provider with mild or moderate persistent asthma and prescribed maintenance ICS treatment and as needed SABA for at least 6 months prior to enrollment.
4. Suboptimal adherence to prescribed maintenance ICS therapy defined as missing at least 2 expected ICS refills in the prior 6 months (>33% missed refills) based on examination of pharmacy records and/or a MARS-A score < 4.5.
5. An ACT score at enrollment greater than or equal to 12 but less than 20 indicating partially controlled or moderately uncontrolled asthma.
6. iPhone or Android smartphone with an active data plan and willingness to use the Hailie® device.

### Exclusion Criteria

1. Relevant comorbid pulmonary diseases including, but not limited to, a diagnosis of chronic obstructive pulmonary disease (COPD), cystic fibrosis, or alpha-1 antitrypsin deficiency.
2. Current use of a biologic medication or investigational treatment for asthma.
3. History of asthma requiring intensive care unit (ICU) admission within the last year.
4. Unwillingness to use or pay for an inhaler that is compatible with the Hailie® sensor (fluticasone propionate or budesonide/formoterol).
5. Any clinically significant abnormalities on physical exam, laboratory testing, or baseline diagnostic testing that the study team believes will make participation in the study unsafe.
6. Patients who do not complete at least 70% of the twice-daily texts during the 2 weeks after screening.

### Subject recruitment plans

We plan to recruit until 50 participants have been randomized, successfully picked up their treatment medication, attached the inhaler sensor, and completed >70% of REDCap questionnaires for 2 weeks.

The recruitment script will be used via phone after potential participants are identified or potential participants will be approached in a clinic. If participants are eligible and willing, twice daily text messages will be sent with their permission to their phone to confirm their ability to complete twice-daily assessments of their asthma control (1 question via REDCap).

#### Randomization method and building

After enrollment and during the first in-person study visit, randomization will then occur as dictated using a 1:1 ratio with random numbers generated using SAS 9.4. This is a pragmatic real-world study. As such the study team and participants will remain unblinded throughout the study period. The patient’s primary asthma provider will be notified of the randomization result and prescribed treatment and their enrollment will be documented in the electronic health record by the study team if they are seen at WUSTL or BJH-affiliated clinics.

#### Risks and benefits

In addition to a detailed consent that will inform all participants of the study risks, the following protections will be put in place:

1. *Confidentiality protections:* All study participants will be assigned a de-identified study ID. A code that matches the participant ID with their name will be kept only in a locked cabinet and/or password-protected computer program per standard procedure and regulations. For the Hailie® device, participants may enter their email and name into the application; however, we will recommend they use a dummy study-provided email, which will be discussed with the patients during the consent process. All study staff will complete and maintain all required training certifications in the responsible conduct of research.

2. *Loss of asthma control:* While the symptom-driven ICS/LABA treatment strategy will only be utilized in participants with mild-to-moderate persistent asthma in whom GINA guidelines recommend this approach, the possibility remains that asthma control could worsen with a change in inhaler strategy in some participants. To mitigate these risks, the Hailie® device will be used for continuous monitoring of participant safety. A trained member of the study team will login to the Hailie® study dashboard at least once every 72 h. Participants who had >8 actuations of ICS/LABA in a day on two consecutive days will be contacted by a study team member. If accuracy of the actuations is confirmed and there are concerns for the safety of the participant continuing in the study, a patient’s providers will be securely notified, and a participant will be considered for removal from the study if deemed appropriate. In addition, a member of the study team will contact the participant every ~8 weeks per protocol to assess for asthma exacerbations and address any participant concerns.

Potential benefits of this research to the participants and others: Potential benefits to participants include education and transition to a new inhaler approach that may be preferrable and more beneficial to them. Providers involved may benefit from education on the latest asthma guidelines and alternative inhaler approaches.

### Early withdrawal of participants

The continued voluntary nature of this study will be discussed at subject enrollment. Furthermore, using the above mechanism for continuous safety monitoring, participants will be monitored by the study team and contacted on an as-needed basis if continued enrollment in the study is not deemed safe.

### When and how to withdraw participants

Contact numbers for the study staff will be provided to participants should they wish to withdraw at any point during the study period.

### Data collection and follow-up for withdrawn participants

If a participant decides to no longer continue in the study or is withdrawn for any reason, they will be invited to return for the second study visit, which can take place either remotely or in-person to assess their asthma control and return their electronic sensor. An addressed and stamped envelope will be sent to their place of residence to return the electronic sensor if they elect to complete this visit remotely or wish to withdraw from the study without completing a final study visit.

### Method for assigning subjects to treatment groups

Participants will be randomized based on the 1:1 randomization schema.

### Preparation and administration of study drug

After randomization at study visit 1, participants will be provided with a new script for their randomized study medications to be filled at their local pharmacy.

### Subject compliance monitoring

The use of the Hailie® device allows for continuous monitoring of participant inhaler actuations and safety monitoring. Inhaler actuation data is sent in real-time to the study team.

### Prior and concomitant therapy

Participants’ inhaler therapies are outlined per the treatment plan. All other concomitant asthma medications will be prescribed at the participant’s primary clinician’s discretion.

### Receiving, storage, dispensing, and return

All study treatments will be prescribed to the participant’s preferred pharmacy. The Hailie® electronic sensors will be stored in a locked cabinet within the research team’s clinical research space and will be given to participants at the in-person first visit or sent to their address after randomization occurs (if the first visit is done remotely).

### Screening for eligibility

Patients will be recruited from WUSTL’s pulmonary and allergy clinics, the study team’s approved database of patients interested in asthma trials, advertisement, or via screening the electronic medical record for eligible participants. Potentially eligible participants will be contacted in a clinic or via the telephone by a member of the study team to inquire about potential interest in the study.

If participants are eligible and willing, twice-daily text messages will be sent with their permission to their phone to confirm their ability and willingness to complete twice-daily assessments of their asthma control (one-item text assessments). Permission for this will be only obtained on the phone. No other interventions whatsoever will take place until written consent is obtained in-person at the first Zoom-based or in-person study visit. Patients who do not complete at least 70% of these one-item text assessments will not be invited for the in-person visit and their participation in the study will be ended.

### Schedule of measurements

Participants will be patients with asthma who meet the inclusion and exclusion criteria and who are able and willing to participate. There will be a total of two in-person or Zoom-based visits outlined below and 3 phone calls during the duration of the study. The phone calls will include a baseline screening and phone calls every 8 weeks during the duration of the study to assess for recent asthma exacerbations.

### Visit 1 (in-person or teleconference option)

At visit 1 (approximately 1–2 h):

1. The patients will be consented using the written consent on paper or via REDCap.
2. Basic demographic and health information will be collected via questionnaire and interview.
3. Questionnaires including the MARS-A questionnaire, medical history questionnaire, ACT, AQLQ, ACQ, IQVIA TSQM, Asthma Self-Efficacy Asthma Scale (ASES), the ICQ-S, the MHLC, and the FSI-10 questionnaire will be administered.
4. The patient will be randomized to continue receiving daily ICS inhalers and SABA (control arm) or to as-needed budesonide/formoterol (treatment arm). For uniformity and to assure the use of a maintenance inhaler compatible with the Hailie® sensor, participants receiving a maintenance ICS other than fluticasone will have their inhaled steroid changed to fluticasone.
5. The Hailie® smartphone application will be downloaded to a patient's phone. If their inhaler is available, the sensor device will be fitted on the inhaler. If the visit is done remotely, the patient will be provided with verbal and written instructions on inhaler sensor installation on their inhaler and we will call them to answer any questions once they fill their new inhaler.
6. We will provide the participant with an Asthma Action Plan to utilize during the study.

### Between visits 1 and 2

1. The participant will continue to use their inhaler and follow their Asthma Action Plan.
2. They will answer two questionnaires a day that take approximately 30 s each. These will be sent via text message with a link to a Health Insurance Portability and Accountability Act of 1996 (HIPAA)-compliant REDCap survey.
3. For patients that completed a virtual initial visit or did not have their inhaler with them at visit 1 if in-person, there will be a call within the first 10 days following randomization to ensure that the participant has set up their electronic sensor adequately and to help troubleshoot any problems that may arise or have arisen.
4. There will be calls at 8 weeks and 16 weeks to assess asthma control, answer questions, and troubleshoot problems.
5. The study team will continue to assess the inhaler use and asthma control.

### Visit 2 (in-person or teleconference option)

At visit 2 (approximately 1–2 h):

1. Participants will be asked questions regarding asthma control over the prior 24 weeks.
2. They will return the sensor and delete the application from their phone (if this visit is Zoom-based they will have been provided an addressed and stamped envelope to return the sensor and shown how to delete the Hailie® application from their phone).
3. Questionnaires including those administered at Visit 1 will be administered again.
4. Participants will be invited to complete an optional brief interview of their opinions regarding their prescribed inhaler approach (this can be done via phone or Zoom up to 2 weeks after this visit if the patient prefers).

### Safety and adverse events

This study is a real-world pragmatic study that involves randomization to one of two inhaler treatment regimens, both of which are FDA-approved and currently advocated for as safe for mild-to-moderate persistent asthma. As such, no adverse events outside of normal clinical care are expected.

### Safety and compliance monitoring

Data and safety monitoring will occur annually by review from the WUSTL IRB. Although no major adverse events are expected to arise that would differ from routine clinical care, the patient’s frequency of inhaler actuations will be monitored in real time. If a patient’s asthma severity increases wherein as-needed use of an ICS/LABA therapy (GINA steps 3–5) is no longer deemed optimal, the patient’s primary clinician will be alerted, the participant will be invited in for their final study visit, and enrollment will be stopped early if needed.

### Study outcome measurements and ascertainment

The primary outcome will be adherence with an inhaler management strategy delineated using the Hailie® inhaler sensor and smartphone-delivered assessments. After the Hailie® sensor is fitted at V1, the Hailie® application will be specifically programmed to deliver morning and evening questions to the participants’ smartphone as shown in Fig. 3. This questionnaire to quickly assess symptoms on a mobile device is identical to what is being used in the multicenter NIH-funded PrecISE trial (ClinicalTrials.gov identifier: NCT04129931). Adherence to the treatment strategy for the maintenance ICS inhaler group will be calculated as the proportion of maintenance ICS actuations per prescription recommendation. Adherence for the symptom-based ICS treatment strategy will be calculated as the proportion of ICS/LABA actuations per recommendation based on self-report of symptoms (definition: ICS/LABA inhaler actuation during a day or night period divided by a period wherein a patient identified symptoms as ≥2 on REDCap smartphone questionnaire).

Secondary outcomes that will be assessed include the frequency of study-defined asthma exacerbations (adjusted for time on treatment), time to first exacerbation, cumulative dose of ICS, occurrence of study-defined severe and non-severe adverse events, and changes in MARS-A questionnaire, ACT, AQLQ, ACQ, IQVIA TSQM, ASES, the ICQ-S, the MHLC, and the FSI-10 questionnaire from baseline to the final study visit. Asthma exacerbations will be defined as an urgent medical visit for acute asthma symptoms, prescription of oral corticosteroids for asthma symptoms, or high use of a rescue inhaler (>16 actuations of SABA or >8 actuations of ICS/LABA within 24 h as assessed via the Hailie® device on two consecutive days). Finally, participants’ degree of satisfaction with their treatment strategy will be assessed using an optional interview at the conclusion of the study.

### Statistical plan

#### Sample size determination and power

Our sample size estimate and power calculation were based on prior studies that asthma patients are adherent with their ICS inhalers 50% (standard deviation [SD]: 37%) of the time and that 80% ICS adherence would result in significant decrements in asthma-related exacerbations and hospitalizations [10, 57]. Assuming a two-sided test of significance with an alpha-error of 0.05, 50 participants in this study (25 per treatment group) would be required to detect an improvement of inhaler adherence from 50 to 80% with a power of 80%. Although improving inhaler adherence to 80% in the symptom-driven ICS/LABA strategy will be difficult, participants will be specifically selected for known maintenance inhaler nonadherence and the maintenance group may have lower ICS inhaler adherence than the estimated 50%.

#### Analysis plan

Study data will be entered into the REDCap electronic data capture tool and undergo quality control checks before transfer into SAS 9.4 for analysis. For the primary analysis, we aim to assess if adherence to an inhaler management strategy differs between treatment groups. Standard descriptive statistics will be used to compare treatment groups. All analyses will compare all participants randomized to each treatment strategy following an intention-to-treat principle followed by per-protocol analyses (i.e., only participants who were randomized to a treatment arm and began using the recommended therapy). Only per-protocol analyses will be done for the primary outcome as it is not possible to do intention-to-treat analyses if the participant does not start recommended therapy in the ICS/LABA group. After appropriate univariable testing is completed, a linear regression model will be constructed and fitted to compare adherence with treatment between groups after adjustment for sex, season of enrollment, and limited other relevant covariates. For secondary outcomes, the change in ACT, AQLQ, ACQ, IQVIA TSQM, ASES, the ICQ-S, the MHLC, and the FSI-10 questionnaire, and cumulative dose of ICS will be similarly evaluated as outcomes using univariable analyses followed by construction of multivariable regression models. Time to first exacerbation will be compared using time-to-event analyses.

#### Missing outcome data

Prior work has demonstrated that the Hailie® electronic sensor has a sensitivity for actuations at 99.9% (97.5% CI 99.7–100%). Thus, limited missing data is expected from real-world inhaler actuations. Some missing data is expected regarding the twice-daily text assessments of asthma control via REDCap. These missing data will be imputed using SAS 9.4. The 2-week period prior to study visit 1 is utilized to identify participants unable to complete these text assessments to limit missing data.

### Confidentiality and security

All study participants will be assigned a de-identified study ID. A code that matches the participant ID with their name will be kept only in a locked cabinet and/or password-protected computer program per regulations. All paper documents with protected health information will be stored in a double-locked location. However, for the Hailie® device, participants will be asked their email and name when setting up the Hailie® application on their smartphone; use of a study-provided dummy email will be recommended. This will be discussed in the informed consent process and no one outside of the approved study team will have access to identifiable data.

#### Part 2: qualitative assessment methods

In accordance with objective 2, a dissemination and implementation (D&I) science conceptual framework was utilized to better understand patients’ and providers’ views of inhaler nonadherence. Semi-structured interviews were guided by and adapted from CFIR.

### Sampling and recruitment

After IRB approval, providers will then be invited to participate in the study through email recruitment, word of mouth, and community outreach. To gather a representative cohort of providers in accordance with qualitative research standards, purposive sampling will be utilized to recruit providers who regularly care for adults with asthma from multiple specialties, including allergy, pulmonary, and primary care medicine. Physicians and advance practice providers of varied experience levels and scopes of practice will be recruited to purposely represent academic and community medicine providers. An overview of study intent and protocol will be provided to interested providers and verbal consent will be obtained prior to beginning the interview. Interviews will be conducted until theme saturation emerged, prospectively estimated at 15–20 interviews.

### Qualitative data collection

A semi-structured interview guide was drafted by an asthma specialist (JGK) and reviewed by an expert in qualitative methodology (ASJ). The interview guide was designed to assess provider views on inhaler nonadherence and the use of symptom-driven inhaled corticosteroid-containing inhalers in real-world clinical practice. See Additional File 1 to view the interview guide used for this study. Interviews will be conducted by graduate students (TCS and VC) with experience in public health and qualitative methodology using semi-structured interview guides. Participants will schedule interviews based on mutual availability and receive study information and consent sheets to review prior to scheduled interviews. In light of the ongoing COVID-19 pandemic and to maximize convenience for all parties, interviews will be conducted virtually via Zoom or phone call. All conducted interviews will be recorded after receiving expressed permission from interviewees.

A brief demographic questionnaire will be verbally administered prior to each interview to collect data on asthma care experience as well as medical training and current practice settings. Interviews are designed to last no more than 60 min in duration and will be recorded using two devices in case of technical failure. Provider anonymity was respected, and no immediate supervisors or other clinical staff were present during the interview. No identifiable information will be collected during interviews for analysis and in the event of disclosure, resulting generated audio transcripts will be de-identified. Field notes will be completed immediately following each interview. See Additional File 2 to view the field note template used for this study. Study compensation included a $50 Amazon gift card for all participants.

### Qualitative analyses

Audio transcripts will be transcribed using a third-party professional medical transcription service. Audio transcriptions will then be imported into NVivo 1.0 (QSR International; Doncaster, Australia) for qualitative analysis. A preliminary codebook was developed inductively based on the interview guide and provider knowledge of asthma care. To ensure codebook integrity, the first four interviews were pilot-tested and reviewed by TCS, VC, and JGK and deductively adapted based on emergent themes.

Following changes to the preliminary codebook, revisions will be proposed and accepted via group discussion before additional coding is undertaken. Subsequently, high interrater reliability will be established before formal coding takes place. All interview scripts will be independently coded by two members of the study team (TCS and VC) and coding discrepancies will be resolved through discussion among the three reviewers. Synchronous review of qualitative coding will ensure that thematic saturation had been achieved and no novel themes emerge thereafter. See Additional File 3 to view the codebook used for this study. Similar techniques will be used for the post-study patient assessments at the conclusion of *Part 1* of the study.

## Discussion

This pragmatic randomized controlled trial attempts to address the common problem of maintenance inhaler non-adherence in asthma by assessing if an alternative treatment methodology may be more beneficial in this group. Inhaler nonadherence remains a significant and often overlooked cause of morbidity in asthma [10, 25, 27, 56–62]. In the US, more than half of the 25 million Americans who have asthma are uncontrolled [1–3, 63]. A lack of asthma control results in >2 million emergency department visits each year and more than $80 billion dollars of annual national cost [64, 65]. Furthermore, the morbidity of asthma disproportionately affects lower socioeconomic status groups [66–68]. While the reasons for poor asthma control are complex, ICS nonadherence, which is estimated to occur in 40–80% of patients, is a major driver of asthma morbidity and is estimated to account for 25% of all asthma exacerbations and 60% of asthma-related hospitalizations [10, 14–16, 29, 69].

Recognizing the immense problem of inhaler nonadherence, international asthma guidelines recently set forth a paradigm-shifting recommendation in inhaler management, and now recommend ICS/LABA inhalers be considered as replacement for SABA for rescue use [30, 31]. With this approach, patients would be exposed to the important anti-inflammatory properties of an ICS when their inhaler is actuated in response to acute symptoms [70]. While this approach has now been well-validated in explanatory clinical trials, it remains unstudied in real-world trials specifically aimed at maintenance inhaler nonadherent patients [33, 34, 36]. Furthermore, patients’ and providers’ views on this inhaler approach have not been fully explored [71]. We postulate that a symptom-driven ICS/LABA treatment strategy may be specifically beneficial as a way of ameliorating the consequences of nonadherence as explored in this study.

This study has numerous novel and innovative elements of clinical trial design. First, this study was designed to be pragmatic, quickly deployable, and minimally intrusive. Second, this study was developed during the COVID-19 pandemic and was designed to have remote options wherein visits could occur via teleconference with Zoom. Thus, in the event of further COVID-19 outbreaks, the study could continue with limited hindrance and minimize disruptions. Finally, this study had a goal of assessing medication adherence (including to an as-needed therapy) in the real world. Partnership with Adherium (NZ) Limited was sought and with their technology, inhaler actuations could be evaluated using the Hailie® device.

One conundrum that came up was how to evaluate adherence to an as-needed therapy. Assessing and comparing adherence between participants using a scheduled ICS therapy and participants using an as-needed ICS therapy is inherently different, though important. Although admittedly imperfect, we developed a strategy of delivering twice daily text messages using Twilio integration into REDCap surveys to assess participant symptoms twice-daily using their smartphone. If a participant was significantly symptomatic, they would be expected to deploy their as-needed therapy. These text messages are one novel way to assess adherence to an as-needed medication through ecological momentary assessments of participants’ asthma symptoms.

## Conclusion

This study is testing a novel inhaler approach in asthma, to ameliorate the detrimental consequences of maintenance inhaler nonadherence, which is a major driver of asthma morbidity and asthma-related healthcare disparities. This trial design has numerous novel design elements including the use of a pragmatic design, use of an FDA-approved sensor to assess inhaler adherence, and strategies to utilize remote visits during the COVID-19 pandemic.

*Study timetable*



## Trial status

This is protocol version 1.4, originally finalized on November 2, 2021, and last revised on April 27, 2022. Recruitment began on December 16, 2021and is estimated to continue until May 30, 2023.

## Supplementary Information

**Additional file 1.** Provider Interview Guide.

**Additional file 2.** Provider Interview Field Note Template.

**Additional file 3.** Provider Interview Codebook.

## Data Availability

The study team will have access to the final de-identified trial datasets. The datasets generated and analyzed during the current study are available from the corresponding author on reasonable request.

## Abbreviations

ACT: Asthma Control Test
ACQ: Asthma Control Questionnaire
AQLQ: Asthma Quality of Life Questionnaire
ASES: Asthma Self-Efficacy Asthma Scale
BJH: Barnes-Jewish Hospital
CFIR: Consolidated Framework for Implementation Research
COPD: Chronic obstructive pulmonary disease
D&I: Dissemination and implementation
FDA: Food and Drug Administration
FSI-10: Feeling of Satisfaction with Inhaler
GINA: Global Initiative of Asthma
HIPAA: Health Insurance Portability and Accountability Act of 1996
ICQ-S: Inhaled Corticosteroid Side-Effect Questionnaire – Brief Version
ICS: Inhaled corticosteroids
ICU: Intensive care unit
IRB: Institutional Review Board
TSQM: IQVIA Treatment Satisfaction Questionnaire for Medication
KASE-AQ: The Knowledge, Attitude, and Asthma Self-Efficacy Asthma Questionnaire
LABA: Long-acting beta-agonist
MARS-A: Medication Adherence Reporting Scale for Asthma
MHLC: Multidimensional Health Locus of Control Scale
EPR-4: National Asthma and Education Program
NIH: National Institutes of Health
PrecISE: Precision Interventions for Severe and/or Exacerbation-Prone Asthma Network
SABA: Short-acting beta-agonists
US: United States
WUSTL: Washington University in St. Louis
