# Supplementary material for: Symptom-driven inhaled corticosteroid/long-acting beta-agonist therapy for adult patients with asthma who are non-adherent to daily maintenance inhalers: a study protocol for a pragmatic randomized controlled trial
Source: Trials. 2022 Dec 5;23:975. doi: 10.1186/s13063-022-06916-3 (PMC9720948; doi:10.1186/s13063-022-06916-3)
Supplement: Supplementary file 1 — Additional file 1. Provider Interview Guide. [file 13063_2022_6916_MOESM1_ESM.docx]

**Additional File 1. Provider Interview Guide**

Interview outline – assessing inhaler adherence and views on as-needed

use of ICS/formoterol (GUIDED BY CFIR Domains)

**PROVIDER SCRIPT**

***Notes for Interviewers:***

***The purpose of the provider interview is to gain a deeper understanding of:***

1. *How providers view the topic of adherence to inhalers in asthma and what providers view as facilitators and barriers to their patients using inhalers.*
2. *If and how providers query their patients regarding inhaler adherence in practice. What providers view as facilitators and barriers to assessing inhaler adherence during a clinical visit.*
3. *Providers’ awareness and views on the latest* ***GINA*** *and* ***EPR-4*** *recommendations in asthma (specifically regarding consideration of symptom-driven ICS-containing inhalers in asthma patients) as well as providers’ views on the facilitators and barriers to utilizing a symptom-driven ICS-containing approach in their patients.*

***Interview Notes:***

- *Interviews can be done in person, on the telephone, or on Zoom (whichever is most convenient for the provider). All COVID-19 guidelines will be followed at all times for interviews and should be carried out with masking or via telephone or Zoom if recommended by current Washington University guidance.*
- *The providers we interview are experts on their own experiences. There are simply no right or wrong answers. All the providers answers help us improve our understanding on the topics we are discussing. Avoid making any judgements about providers’ actions or beliefs.*
- *Ideally, let the provider talk for as long as they want on any question without interruption. Silence and pauses are just fine. Do not immediately jump in to fill pauses.*
- *When probing a provider further, restate the provider’s original comment to show that you’re engaged in the interview.*

***Helpful Questions and Probes to Consider during a Provider Interview:***

- *Why do you think that happens?*
- *Tell me a little more about what you just said…*
- *Can you give me an example of….?*
- *What happens next?*

I**NTRODUCTION NOTES:**

1. First, be sure **thank** the provider for taking the time to talk with you.
2. Introduce who you are: what your name is and what your role on this qualitative project is.
3. Re-iterate that their participation is completely voluntary.
4. Introduce what the purpose of this project is in simple terms:
5. Tell the provider you expect the entire interview to take 30-45 minutes but that it can be stopped at any time for any reason. (Compensation for their time will be provided even if they decide to stop the interview early).

A sample introduction could be something like:

“You are Dr. *** ? Good to meet you.”

*“Thanks for taking your time to talk with me today. I’m grateful and know that you are busy. My name is ***. I am a research assistant who is working with this study led by Dr. Krings, an Assistant Professor of Pulmonary Medicine at Washington University. We’re trying to better understand how asthma patients take their inhalers - in your experience. Additionally, we’re interested in your awareness and views of some recent asthma recommendations.”*

*“We’ll ask you some questions and we simply ask for your honesty – there are no right or wrong answers. Obviously, the more open and honest you are in your answers, the better, but also know that if I ask you a question and you prefer not to answer, that is totally okay too. We estimate this entire interview will take about 30-45 minutes.” Okay?*

**INFORMED CONSENT**

Before we go any further, did you receive and review the electronic consent form?

*(If asked, can remind patient: there may be no benefits to participating in today’s interview. The only foreseeable risks are unexpected loss of confidentiality, embarrassment or discomfort from discussing individual professional practices, or recounting distressing scenarios with patients. As a token of appreciation for your time, you will receive a gift card to Amazon.com in the amount of $50).*

Do you consent to this interview?

- Yes *(Proceed with interview)*
- No *(End Interview. State: “We appreciate your time. Thank you for considering participation in this project.”)*

**RECORDING**

“If it is okay with you, we would like to **record and then transcribe this interview**. We will be recording today’s interview only so that we have an accurate record of what you say. We’re not trying to collect anything identifiable about you. If you say something during today’s interview that will identify you, we will remove it from the transcript. Only study personnel will have access to the audio files and transcripts, and they will be stored on secure servers. Is recording this interview, okay?

*(If yes, start the recorder now. If no, stop the interview now, and thank the provider for his or her time)*

**BEFORE PROCEEDING FURTHER:**

- “Great. Do you have any questions for us before we begin the interview?” Answer questions

**“To begin with, I’d like to ask some basic things about you and your practice…”**

***BASIC QUESTIONS ON CLINICAL PRACTICE:***

- “Are you a physician (*such as an MD, DO, or equivalent*) or advanced care provider (*such as an NP or PA*)?”
- “Do you normally take care of adults with asthma, children with asthma, or both?”
- “Do you primarily work in a primary care office, urgent care setting, pulmonology office, or allergy office?”
- “Where do you primarily see patients? Is your practice part of a larger group such as BJC, SLU, or Mercy?”
- “How many patients would you estimate you take care of with asthma in a normal week?”
- “How many years have you been taking care of patients with asthma?”
- “How do you learn about new recommendations in medicine? (example reading guidelines, conferences, colleagues, all of the above).”
- “What are the barriers to learning new guidelines in medicine you’ve noticed? To staying up things?”

**“That makes sense. Thanks…”**

***QUESTIONS ON INHALER ADHERENCE AND PROCESSES CLINICIANS USE FOR ASSESSING INHALER ADHERENCE:***

- “As we stated at the beginning, we’re trying to better understand how you think your patients use their inhalers **in reality**, and how you assess for inhaler adherence during your routine clinical visits.”
- “Let’s think of a typical case for you. If you need help imagining a case, let me give you a hypothetical example…let’s say you’re seeing a 40-year-old woman with mild persistent asthma who you’ve previously prescribed a maintenance ICS inhaler to (like beclomethasone [QVAR]”
  - “What does that encounter look like? What kind of questions would you ask her during a clinic appointment?”
  - “Do you ask about maintenance inhaler adherence at every clinic visit?“
  - “How do you assess if a patient like this one is adherent to their inhalers you’re prescribing?”

**Be sure they answer things like…**

- - - **Asking?**
    - **Looking in medical record to see refills?**
    - **Using a validated questionnaire that assess adherence?**
  - “Tell me more about why you do or don’t assess inhaler adherence more often?”
  - “Do you think patients are generally truthful with you about inhaler adherence?”
  - “Do patients express concern to you about being exposed to an inhaled corticosteroid on a regular basis (in an ICS)?”
  - “What do you do if your patient is non-adherent to their maintenance inhaler?”
- “Let’s think about an electronic smart sensor that goes on top of an inhaler and collects data on when patients are using their inhaler. Do you think that would be helpful to you?
- “How would you want that adherence data presented (in the EMR, some other way)?”
- “Any other thoughts you’d like to share with us on the topic of inhaler adherence?”

***QUESTIONS ON LATEST GINA AND EPR-4 RECOMMENDATIONS FOR SYMPTOM-DRIVEN USE OF ICS-CONTAINING INHALERS:***

(This part of the interview may vary based on whether the provider has knowledge of the latest GINA and EPR-4 recommendations. Some will likely have knowledge. Some likely will not. Interviewing both groups of providers is helpful and informative here. If they are not aware of the latest guidelines, take a few minutes and explain it to them – don’t tell people your views or the pros and cons of the recommendations – our hope is to learn more about what they think. They are the clinician experts).

- “I’d now like to talk about something a little bit different”
- “Are you aware that some groups (like GINA) in asthma are discussing how ICS-containing inhalers (like budesonide/formoterol, or Symbicort) can be used on a rescue basis?”
  - If they answer no on awareness: “This is an approach where the provider – like you – would prescribe Symbicort to people with mild asthma for rescue use – in place of their albuterol. What do you think of that?”
  - If they say, I’d need to know more…”the rationale is that people who just use an albuterol inhaler on a rescue basis still have severe exacerbations and may benefit from being exposed to an ICS. In the trials, people that got a rescue ICS/LABA inhaler actually did better than those that got a rescue short-acting beta agonist only as far as exacerbation risk in mild asthma (SYGMA trial – in New England Journal of Medicine)”
- “What do you think about that? Do you have any views on that? Pros cons?
  - “If you were already familiar with the recommendation for consideration of an ICS-containing reliever therapy, do you have any opinions on the quality of the evidence backing this up? It’s okay to say I don’t know”
- “Do you use budesonide/formotoreol (or Symbicort) in your practice on a rescue basis? Why do you? Or why do you not use it?”
  - “If **YES**, when do you utilize this approach? For what patients? Why? What have you observed as the benefits of this approach? What have you observed as the disadvantages of this approach? What have you observed are the barriers to this approach?”
  - “If **NO**, would you consider utilizing symptom-based ICS/formoterol in your practice? What would be the pros and cons you can think of?”
- “Some of the latest asthma recommendations involve “either or” choices in treatments. For example, in mild persistent asthma, one could consider either “maintenance ICS inhalers and albuterol as needed **OR** no maintenance ICS inhaler and a combined ICS/beta agonist as needed.” Do you regularly involve patients in these decisions when treatments are “either or”? Why or why not?”
- “If you learned that a patient of yours was not taking their maintenance ICS inhaler most of the time and only using a short-acting beta agonist (like albuterol) do you think it would be worthwhile to consider changing their reliever therapy option to an ICS-beta agonist so they have some exposure to an ICS? Why or why not? Is that a good idea?”

Almost done…

**FACILITATORS AND BARRIERS TO USE OF *SYMPTOM-DRIVEN USE OF ICS-CONTAINING INHALERS***

- “We’ve now been talking about symptom-driven use of ICS-containing inhalers for a while.
- “Do you think you have the ability to start utilizing this approach now? Why or why not?”
- I do not know if you this: At the moment, the use of symptom-driven ICS-containing inhalers is advocated by expert groups like GINA. But currently ICS-containing inhalers are not actually FDA approved specifically as a reliever therapy. Does that make you reconsider your use of this approach (i.e. the FDA labeled indication)?”
- “Now, if you decide to utilize ICS-beta agonist inhalers on a symptom-driven basis, some providers write the script as BID standing (twice daily every day) ...while telling a patient to really only use a medication on an as needed basis (since it is technically off label from an FDA-perspective as a reliever therapy). Do you consider this concerning? Or a major barrier to utilizing this approach? “
- Another approach along this thought process, is having patients take a short-acting beta agonist and ICS inhaler at the same time when they are having symptoms (rather than a combined ICS-formoterol inhaler)? Do you think this would be feasible for most of your patients?

**CONCLUSION**

- “Do you have any final thoughts you want to share with us? Do you have any other recommendations for us when we’re thinking about maintenance inhaler adherence and the use of symptom-driven ICS inhalers?”
- “After this interview you should receive an email in about an hour linking you to a REDCap database. This will collect some information from you so you can receive your payment. It will also ask if you have any other providers you’d recommend we interview”
- “Thanks again. Please reach out if we can answer any questions in the future.”
