# Supplementary material for: Symptom-driven inhaled corticosteroid/long-acting beta-agonist therapy for adult patients with asthma who are non-adherent to daily maintenance inhalers: a study protocol for a pragmatic randomized controlled trial
Source: Trials. 2022 Dec 5;23:975. doi: 10.1186/s13063-022-06916-3 (PMC9720948; doi:10.1186/s13063-022-06916-3)
Supplement: Supplementary file 2 — Additional file 2. Provider Interview Field Note Template. [file 13063_2022_6916_MOESM2_ESM.docx]

**Additional File 2. Provider Interview Field Note Template**

**Participant Name:**

**Interviewer Name:**

**Date:**

**Location of Interview:**

**Time of Interview:**

**Observational notes** (Anything you observe about the participant, other people, the place or the environment in which the interview takes place. Any information that might influence the information you gather): Examples • Description / sketch of the interview location Was it hot? Cold? Noisy? Not very private? • Whether or not other people are present during the interview • Observations about the participant: Are they nervous? Comfortable? Shy? Anxious? Do they seem to understand the questions?


**Methodological notes (Comments on the process of the actual interview):**

Examples • Comments about the research protocol Did participant understand the information and informed consent? Are you satisfied that the participant’s privacy is respected? • Comments about the interview guide: Changes in the order of the questions Topics or themes that were particularly important in the interview Difficulties with certain questions Length of interview Interruptions in the interview


**Theoretical notes (Refer to the objectives of the interview):**

Examples • Comments on how this interview begins to answer the research questions • Common themes or experiences across interviews • New questions that are raised by the interview • Specific words or phrases that may be meaningful (insider language) • Summaries of conversations


**Personal notes (How you felt during the interview – other reflections)**

Examples: • “I lost track of the conversation a few times and I think I missed a few opportunities to follow-up on important things the participant said. Pronouncing words.  I got the feeling that the participant was holding back.”
