# Supplementary material for: Symptom-driven inhaled corticosteroid/long-acting beta-agonist therapy for adult patients with asthma who are non-adherent to daily maintenance inhalers: a study protocol for a pragmatic randomized controlled trial
Source: Trials. 2022 Dec 5;23:975. doi: 10.1186/s13063-022-06916-3 (PMC9720948; doi:10.1186/s13063-022-06916-3)
Supplement: Supplementary file 3 — Additional file 3. Provider Interview Codebook. [file 13063_2022_6916_MOESM3_ESM.docx]

**Additional File 3. Provider Interview Codebook**

| **Code** |  | **Definition, Inclusion, Exclusion Criteria** | **Specific Example** |
| --- | --- | --- | --- |
|  | **Innovation Characteristics** |  |  |
| **1. Evidence** | **Evidence Strength & Quality** | Definition: Provider’s perceptions of the quality and validity of evidence supporting as-needed ICS/LABA inhalers.  Inclusion Criteria: Include statements regarding awareness of evidence and the strength and quality of evidence, as well as the absence of evidence or a desire for different types of evidence. |  |
| 1.01   Evidence_overall |  | Definition: Provider gives some views about the evidence but does not explicitly express whether they believe it is good or bad. |  |
| 1.02   Evidence_good |  | Definition: Believes evidence for recommendation to use this inhaler approach is good. | “This is based on multiple RCTs. I believe the evidence is good.” |
| 1.03   Evidence_nogood |  | Definition: Believes evidence for recommendation to use this inhaler approach is not adequate, mediocre, not good. |  |
| 1.04   Evidence_dontknow |  | Definition: Unaware of the underlying evidence to use this inhaler approach. | “I don’t really know. I’ve heard about this approach and the guidelines but I don’t know the evidence itself.” |
| **2. Design Quality & Packaging** | **Design Quality & Packaging** | Definition: Perceived excellence in how the intervention is bundled, presented and assembled. |  |
| **3. Radvantage** | **Relative Advantage** | Definition: Provider’s perception of the advantage of implementing this inhaler approach versus standard approach.  Inclusion Criteria: Include statements that demonstrate the new inhaler approach is better (or worse) than traditional inhaler therapy.  Exclusion Criteria: Exclude statements that demonstrate a strong need for the new inhaler approach and/or that the current situation is untenable and code to [Tension for Change](http://cfirwiki.net/wiki/index.php?title=Tension_for_Change). |  |
| 3.01   Radvantage_good |  | Definition: Overall there is an advantage to using this inhaler approach. |  |
| 3.02   Radvantage_nogood |  | Definition: Overall there is not an advantage to using this inhaler approach. |  |
| 3.03   Radvantage_dontknow |  | Definition: Unaware of what inhaler approach is more advantageous. |  |
| **4. Complex** | **Complexity** | Definition: Perceived difficulty of utilizing new inhaler approach in clinic, reflected by radicalness, intricacy, and number of steps in each approach.  Inclusion Criteria: Code statements regarding the complexity of the inhaler approach itself.  Exclusion Criteria: Exclude statements regarding the complexity of implementation and code to the appropriate CFIR code. |  |
| 4.01   Complex_yes |  | Definition: Believes overall new inhaler approach is complex. | “This new inhaler approach seems too complex to explain. They can just keep doing what they’re doing.” |
| 4.02   Complex_no |  | Definition: Believes overall new inhaler approach is not/less complex than traditional therapy. | “This new inhaler approach is so easy to explain.” |
| **5. Cost** | **Cost** | Definition: Costs of the new inhaler approach and costs associated with implementing the innovation (cost of learning how to use).  Inclusion Criteria: Include statements related to the cost of the innovation and its implementation. |  |
| 5.01   Cost_overall |  | Definition: Comprehensive code relating to cost. |  |
| 5.02   Cost_higher |  | Definition: Provider believes new inhaler approach may be more expensive for patients. | “I am concerned Symbicort won’t be covered by their insurance and they will pay more.” |
| 5.03   Cost_lower |  | Definition: Provider believes new inhaler approach may be cheaper for patients. | “I believe patients will pay less as they need to get less inhalers filled with this new approach.” |
| 5.04   Cost_noidea |  | Definition: Provider endorses of a feeling of not knowing what inhaler approach is more cost-effective for patients. | “I just have no idea what people will pay.” |
|  | **Outer Setting** |  |  |
| **6. Peer** | **Peer Pressure** | Definition: Provider conveys pressure to implement this inhaler approach because others in their field are, their colleagues are. | “I don’t really know. I guess I feel I have to use this since my colleagues, the pulmonary and allergy experts are.” |
| **7. External Policy & Incentives** | **External Policy & Incentives** | Definition: A broad construct that includes external strategies to spread innovations including policy and regulations, recommendations and guidelines, pay-for-performance, collaboratives, and public or benchmark reporting.  Inclusion Criteria: Include descriptions of guideline (how good they are), FDA policy, insurance policy.  Exclusion Criteria: Do not include opinions on quality of evidence (that is coded under evidence above) that underlies FDA decisions, reimbursement decisions, etc. |  |
| 7.00   FDA_overall |  | Definition: Provider’s views on the FDA with relation to this approach. |  |
| 7.01   FDA_effect |  | Definition: Decision of FDA to not fully approve this approach has an effect on their decision how or if to use this approach. |  |
| 7.02   FDA_noeffect |  | Definition: Decision of FDA to not fully approve this approach has no effect on their decision how or if to use this approach. | “Whether or not this approach is FDA approved does not affect my decision to prescribe.” |
| 7.03   Guideline_effect |  | Definition: Decision of guidelines (GINA and EPR-4) to endorse this approach has an effect on their decision to use this approach. |  |
| 7.04   Guideline_noeffect |  | Definition: Decision of guidelines (GINA and EPR-4) to endorse this approach has not affected their decision to use this approach. | “I end up making my own decisions. I can’t be aware of every guideline. I have experience and like to make my own decisions – not just listen to whatever some group is saying.” |
| 7.05   Insurance_overall |  | Definition: Provider mentions insurance with relation to this approach. |  |
| 7.06   Insurance_effect |  | Definition: Decision of insurance to not fully approve this approach and my awareness if they will pay has an effect on my decision to use this approach. | “Will insurance even pay for this approach? I don’t know. That makes me not want to prescribe as-needed Symbicort.” |
| 7.07   Insurance_noeffect |  | Definition: Decision of insurance to not fully approve this approach and my awareness if they will pay has no effect on my decision to use this approach. |  |
|  | **Inner Setting** |  |  |
| **8. Climate** | **Implementation Climate** | Definition: The absorptive capacity for change in a provider’s clinic, and the extent to which use of new guidelines such as this one will be rewarded, supported, and expected within their organization.  Inclusion Criteria: Include statements regarding the general level of receptivity to implementing the innovation. |  |
| 8.01   Climate_change |  | Definition: Climate of clinic is one to always enact the latest guidelines, change practice constantly. | “Our clinic culture is to always stay up and enact whatever the latest guidelines are.” |
| 8.02   Climate_nochange |  | Definition: Climate of clinic is not one to enact the latest guidelines, change practice constantly. |  |
| **9. Tension** | **Tension for Change** | Definition: The degree to which stakeholders perceive the current situation (non-adherence) as intolerable or needing change.  Inclusion Criteria: Include statements that demonstrate a strong need for a new inhaler approach and/or that the current situation is untenable, e.g., inhaler adherence is dismal.  Exclusion Criteria: Exclude statements that demonstrate the innovation is better (or worse) than existing programs and code to [Relative Advantage](http://cfirwiki.net/wiki/index.php?title=Relative_Advantage). | “Maintenance inhaler use is so bad in some patients. Might as well try something different.” |
| **10. Priority** | **Relative Priority** | Definition: Individuals’ shared perception of the importance of this approach within their practice. |  |
| 10.01   Priority_high |  | Definition: Individuals’ shared perception of the importance of this approach within their practice is important. | “Asthma is a huge piece of what we do. It is an important part of my day. |
| 10.02   Priority_low |  | Definition: Individuals’ shared perception of the importance of this approach within their practice is not important. | “I’m just too busy with primary care needs. I can’t assess adherence on a daily basis or think about different approaches for different people.” |
| **11. Access to Knowledge and Information** | **Access to Knowledge and Information** | Definition: Ease of access to digestible information and knowledge about the intervention and how to incorporate it into their practice. |  |
| **12. Available Resources** | **Available Resources** | Definition: The level of resources dedicated for implementation and on-going operations, including money, training, education, physical space, and time. |  |
| **13. Characteristics** | **Characteristics of Individuals** |  |  |
| 13.01   Char_adult |  | Definition: Provider takes care of adults. |  |
| 13.02   Char_children |  | Definition: Provider takes care of children. |  |
| 13.03   Char_both |  | Definition: Provider takes care of both adults and children. |  |
| 13.04   Char_physician |  | Definition: Provider is a physician. |  |
| 13.05   Char_APP |  | Definition: Provider is an NP or PA. |  |
| 13.06   Char_pulm |  | Definition: Provider primarily identifies as working in a pulmonary office. |  |
| 13.07   Char_allergy |  | Definition: Provider primarily identifies as working in an allergy office. |  |
| 13.08   Char_PCP |  | Definition: Provider primarily identifies as working in a primary care office. |  |
| 13.09   Char_clinic_large |  | Definition: Provider primarily identifies as part of a large office (BJC, Mercy, etc.). |  |
| 13.10   Char_clinic_small |  | Definition: Provider primarily identifies as part of a small office. |  |
| 13.11   Char_asthma_number |  | Definition: Provider discusses how many asthma patients they care for. |  |
| 13.12   Char_experience |  | Definition: Provider discusses their experience level (number of years working clinically). |  |
| **14. Knowledge** | **Knowledge & Beliefs New Inhaler Approach** | Definition: Individuals’ attitudes toward and value placed on the inhaler approach, as well as familiarity with facts, truths, and principles related to the innovation.  Exclusion Criteria: Exclude statements related to familiarity with evidence about the innovation and code to [Evidence Strength & Quality](http://cfirwiki.net/wiki/index.php?title=Evidence_Strength_%26_Quality). |  |
| 14.01   Knowledge_good |  | Definition: Provider knows a lot about this new inhaler approach. | “Oh yes, I know a lot about it.” (Irrelevant whether they use it.) |
| 14.02   Knowledge_nogood |  | Definition: Provider knows very little or nothing about this new inhaler approach. | “Yeah, I’ve vaguely heard about it.” |
| 14.03   Knowledge_intermediate |  | Definition: Provider knows somewhat about this new inhaler approach. |  |
| 14.04   Views_positive |  | Definition: Provider views this new inhaler approach favorably. |  |
| 14.05   Views_negative |  | Definition: Provider views this new inhaler approach negatively. |  |
| 14.06   Views_dontknow |  | Definition: Provider is unsure of their views. |  |
| **15.  Self-efficacy** | **Self-efficacy** | Definition: Individual belief in their own capabilities to execute courses of action to use approach. |  |
| **16.  Stage** | **Individual Stage of Change** | Definition: Characterization of the phase an individual is in, as they progress toward skilled, enthusiastic, and sustained use of the inhaler approach. |  |
| 16.01   Stage_never |  | Definition: Provider who never uses this approach. |  |
| 16.02   Stage_rarely |  | Definition: Provider who rarely uses this inhaler approach. | “I’ve tried it in 1-2 patients.” |
| 16.03   Stage_frequently |  | Definition: Provider who frequently uses this inhaler approach. | “Oh yes. I use it all the time.” |
| **17. Personal Attributes** | **Other Personal Attributes** |  |  |
| 17.01   Personal_paternalistic |  | Definition: Provider who is paternalistic in their approach. |  |
| 17.02   Personal_collaborative |  | Definition: Provider who is collaborative in their approach. |  |
| **18. Process** | **Process** |  |  |
| **19. Leaders** | **Leaders** | Definition: Individuals in an organization that have formal or informal influence on the attitudes and beliefs of their colleagues with respect to implementing the innovation.  Inclusion Criteria: People in their clinical setting (not guideline bodies) have talked about this approach, caused them to change their approach. | “Oh yes, I heard one of our asthma doctors discuss this. It made me think about it.” |
| 19.01   Leaders_champion |  | Definition: Individuals who dedicate themselves to supporting, marketing, and ‘driving through’ an [implementation]”, overcoming indifference or resistance that the intervention may provoke in an organization. |  |
| 19.02   Leaders_opinion |  | Definition: Individuals in an organization who have formal or informal influence on the attitudes and beliefs of their colleagues with respect to implementing the intervention. |  |
| **20. Adherence** | **Asking about Adherence** | Definition: Provider’s frequency of asking about adherence. |  |
| 20.01   Adherence_ask_freq |  | Definition: Provider’s frequency of asking about adherence is always or frequently. |  |
| 20.02   Adherence_ask_rarely |  | Definition: Provider’s frequency of asking about adherence is rarely or never. |  |
| 20.03   Adherence_ask_sometimes |  | Definition: Provider’s frequency of asking about adherence is intermediary. |  |
| 20.04   Adherence_ask_quality |  | Definition: Provider’s response speaks to the quality of the way they assess adherence. |  |
| Adherence_method |  | Definition: Method provider uses to inquire about adherence. |  |
| 20.05 Adherence_method_  verballyask |  | Definition: Provider verbally asks patient to assess adherence. |  |
| 20.06   Adherence_method_ questionnaire |  | Definition: Provider uses a validated questionnaire to assess adherence. |  |
| 20.07   Adherence_method_ medrecord |  | Definition: Provider uses a review of medical records to assess adherence. |  |
| 20.08   Adherence_method_sensor |  | Definition: Provider uses an electronic inhaler sensor to assess adherence. |  |
| 20.09   Adherence_truth |  | Definition: Provider’s assessment of the truthfulness of patients regarding inhaler adherence. |  |
| **21. Steroid / Sensor** |  |  |  |
| 21.01   Steroid_concern |  | Definition: Provider’s assessment that patients are concerned about ICS exposure. |  |
| 21.02   Sensor |  | Definition: Provider discusses their views on inhaler sensors. |  |
| 21.03   Sensor_data |  | Definition: Provider discusses their views on inhaler sensor data should be presented. |  |
| **22. Miscellaneous Codes** |  |  |  |
| 22.01   Misc_quote |  | Definition: Information deemed or designated as quoteworthy. |  |
| 21.02   Misc_other |  | Definition: Information not sufficiently captured with other codes. |  |
